# COVID-19 post-pandemic reflections from sub-Saharan Africa: what we know now that we wish we knew then

**DOI:** 10.1016/j.puhip.2024.100486

**Published:** 2024-02-29

**Authors:** Obinna O. Oleribe, Simon D. Taylor-Robinson, Andrew W. Taylor-Robinson

**Affiliations:** a Nigerian Institute for Medical Research, Lagos, Nigeria; b Best Health Consult Limited Liability Company, Orange, CA, USA; c Department of Medicine, Busitema University, Mbale, Uganda; d Department of Public Health, Busitema University, Mbale, Uganda; e Department of Surgery and Cancer, Imperial College London, St Mary's Hospital Campus, London, UK; f College of Health Sciences, VinUniversity, Hanoi, Viet Nam; g Center for Global Health, Perelman School of Medicine, University of Pennsylvania, Philadelphia, PA, USA

**Keywords:** COVID-19, SARS-CoV-2, Infectious disease, Pandemic, Prevention, Control, Preparedness, Sub-Saharan Africa

## Abstract

The commonly heard aphorism about history repeating itself suggests an endless cycle of recurring events. However, George Santayana offered a similar sentiment when he said, "Those who do not learn from history are doomed to repeat it". This emphasises that the responsibility for the recurrence of events lies not with history itself, but with humanity. It underscores that if we desire change, it is our responsibility to initiate it, rather than attributing it to external forces such as fate, luck, or time. With this thought in mind, here we offer a narrative view from sub-Saharan Africa, focusing primarily on our own experiences in Nigeria and Uganda, on what harsh lessons can be learnt from the COVID-19 pandemic regarding emergency preparedness to respond effectively to the next major infectious disease outbreak. Four strategies are suggested, the implementation of which may contribute substantially to safeguarding against an experience similar to the catastrophic public health, social and economic costs borne by African nations during COVID-19 and in its immediate aftermath.

## 1 What this study adds

• The article rigorously examines insights gained from the COVID-19 pandemic in sub-Saharan Africa, specifically focusing on the experiences in Nigeria and Uganda.
• It explores critical aspects of pandemic management and provides recommendations for future infectious disease outbreaks.
• It emphasises separating medical practice from business interests and giving priority to patient care through training of frontline professionals, improvement of healthcare infrastructures, and networking for ready exchange among countries on the continent.

## 2 Implications for policy and practice

• The article effectively advocates for the separation of science from politics and emphasises the importance of evidence-based decision-making by involving public health professionals.
• It underscores the need to differentiate proven public health practices from untested behaviours and stresses the importance of culturally sensitive prevention strategies.
• It highlights that policies of social and cultural innovation are required that make nations ready to respond to catastrophic events on their territory through access to data, communities of practice, co-creation, reflection and inclusion.

## 3 Introduction

Much has been published on COVID-19 from all parts of the world [[1], [2], [3], [4]]. We have also written several manuscripts on the epidemiology of the pandemic, people's perspectives, lessons learned, and why Africa was relatively spared from the worst of the pandemic, even after recognising that case ascertainment and reported mortality rates across the continent were far from complete [[5], [6], [7], [8], [9], [10]]. However, there is still considerably more on which the global health community can reflect as the world transitions into the post-pandemic era.

While there are still new infections and deaths, the 4-year-long COVID-19 pandemic appears to be drawing to a close. The toll exacted on humankind is more than 6.9 million recorded deaths, with unquantifiable hardship, and ongoing complications of long-COVID [[11], [12], [13]]. At the peak of COVID-19, several policies were made that impacted global and local travel, human relationships, work practices, and people's social lives. The frequently strict enforcement of measures to control community transmission of the causative SARS-CoV-2 virus hit the poor, marginalised and underserved members of each society the hardest, often with long-term psychological and physical consequences that were not anticipated [[11], [12], [13]]. New operating procedures were developed that changed the way infectious diseases were monitored, managed, and mitigated [[14], [15], [16]]. Novel non-pharmaceutical interventions and vaccines were fast-tracked into development and manufacture to curtail the worst effects of COVID-19 [14]. Nations invested heavily in vaccine production, some prepaying for millions of vaccine doses even before they were tested and declared effective by any regulatory agency [16].

Drawing on the West and East African examples of Nigeria and Uganda, the national governments made several promises – some of which they were not able to keep. Frequently, front-line workers who failed to keep to the hastily approved policies and procedures were punished or relieved of their posts [6]. Some individuals (including the first author) missed their international flights because of late or expired COVID-19 rapid antigen test results. Governments made money from travel corridor mandatory testing and screening, and new businesses emerged to exploit the benefits of the pandemic policies and programs [7].

Africa has plentiful historical experience of containing serious infectious diseases, such as Ebola outbreaks in recent times and the long-forgotten “Spanish” influenza pandemic of 1918–1919. Yet, there is no doubt that in the rush to counter the impending COVID-19 disease wave, the public health lessons of the past were seemingly ignored. For instance, many hastily developed containment policies had little scope for public engagement and were often adopted without expert medical advice [17,18]. We ask what, therefore, has sub-Saharan Africa learned from this pandemic that could help us manage future epidemic and pandemic infectious diseases outbreaks more effectively and efficiently? Here are our observations and recommendations.

## 4 Separating science from politics

In responding to COVID-19 initially, the governments of some nations, including many in Africa, moved into the driver's seat to direct the pandemic control program and process, even though they did not have the right qualifications and credentials to make scientific decisions or statements. Some made promises on vaccine production, effectiveness, efficacy, and use without allowing the right vaccine and therapeutics development processes to be followed [[19], [20], [21]]. This fueled all types of conspiracy theories and resistance to vaccine uptake across all nations of the world [22,23]. When scientists later made statements or released study reports predicated on recent research discoveries, their findings were doubted as people's views were already biased by political interventions earlier in the pandemic [7].

In their desire to reopen societal systems as quickly as possible, thereby preventing impending economic collapse, governments also made vaccination and other pandemic control practices mandatory, infringing human rights and restricting individual freedom of choice [24]. In Nigeria, for example, governmental and public organisational staff were mandated to be vaccinated or otherwise lose their jobs. The use of face masks, social isolation, and quarantine were all made compulsory in many jurisdictions. Travel was prohibited and social events were canceled. Places of worship, schools, businesses, restaurants, and shopping malls were all closed [5].

Individuals who valued their freedom resisted this political intrusion – even if it was just for the sake of protecting and preserving their freedom of choice. Vaccine refusal and hesitancy surged to an all-time high and conspiracy theories soared across social media [25]. Many denied the effectiveness of different vaccines, challenged their development processes, and saw the entire process as fraudulent and money-making for biotech and pharmaceutical companies who exploited pandemic-fueled societal fear to make untold profits in the return on investment [26].

Moving forward, it is our perspective from sub-Saharan Africa that governments should steer clear from making uninformed statements on infectious disease outbreaks [17,18], resisting political pressure to attempt to control what they do not understand [[19], [20], [21]]. Rather, when aiming to prevent or eliminate future epidemics and pandemics, evidence-based decisions should be left in the jurisdiction of suitably trained and qualified public health professionals. Much could have been learnt during the COVID-19 pandemic from past experiences of Ebola and Lassa fever outbreak responses, which greatly advanced expertise on pathogen containment across the continent. However, instead of setting up independent advisory commissions to define mitigation policies, the knowledge of regional experts was largely overlooked in favour of World Health Organization directives that did not consider local maxims or community wisdom.

## 5 Separating medical practice from business

With COVID-19, there was considerable business interest in the management and control of the pandemic [27]. Entrepreneurs made millions to billions of dollars from the development and marketing of face masks, hand sanitisers, antiseptics, ventilators, and personal protective equipment in the first 18 months of the pandemic, and from the distribution of vaccines and therapeutics in the subsequent period [28]. Although COVID-19 was projected to devastate economies, this proved not to be the case for many high- and middle-income nations as immediate negative impacts were short-lived [29,30]. While these resources were greatly needed to translate policies into practices across different global regions, the emphasis on profitability and gains by manufacturers fueled societal outbursts, anger, and revolt.

During this time of great uncertainty, insurance companies in some African countries continued collecting premiums from individuals even though hospitals and health institutions were off limits to those who did not have COVID-19 and governments were paying for treatment of those infected with SARS-CoV-2 and admitted for care [31]. While this situation was by no means universal across the continent, one of the authors heard a leading insurance company executive boast that they made more money during COVID-19 than in any other period in their history. The concern is that if this apparent greed is not addressed, those insuring the use of the commercial healthcare sector may seek further monetary gains for their organisations in the face of future public health threats.

With this objective in mind, should insurance companies be asked to refund all the premiums collected during COVID-19 to participating individuals and recommence premium collections only at the conclusion of this pandemic era? From the perspective of justice and fairness in managing pandemics, this would be an equitable, if not expected outcome [26]. Otherwise, in those countries where this was prevalent, we will be tacitly and implicitly encouraging “double dipping” by insurance companies that during the pandemic kept collecting premiums every month while still allowing governments to foot the bill for treatment of all clinical cases when COVID-19 management made up over 90% of healthcare costs in developed and developing countries alike. In sub-Saharan Africa, this redirection of resources drained national budgets for public health, leading to reduced allocations for prevention, control and therapy of other notable infectious diseases, such as malaria, HIV, tuberculosis and schistosomiasis. This resulted in increased morbidity and mortality from easily preventable diseases [32]. One could argue that insurance companies should insure themselves against losses incurred in future pandemics, so that the public and governmental sectors do not carry unduly the burden of any increase in expenditure.

## 6 Separating proven public health practices from untested behaviours

Consequent to the escalating global death toll due to COVID-19, there was a pressing need to institute a range of non-pharmaceutical interventions to curb aerosol and droplet transmission of SARS-CoV-2 [14]. This led to the promoting of handwashing and the wearing of face masks, each a tried and tested public health preventive practice, alongside several other previously untested practices like elbow bumping and physical distancing [16]. In addition, as the pandemic deepened, masks and respirators also evolved from high-quality clinically-tested versions to a varied assortment of facial coverings produced from all manner of fabrics with little or no effectiveness and efficiency testing [16]. The unregulated manufacture of poorly protective masks flooded the global online market, the popularity of what soon became fashion accessories boosted across social strata. People began designing and selling poorly fitting masks handmade from unsuitable materials as there were gullible individuals ready with cash to buy and use, even if the products were ineffective [33].

The production of hand sanitisers and personal protective equipment was also unregulated during the pandemic due to overwhelming demand. Quality and effectiveness were sacrificed at the expense of quantity and access. For the vast majority of such nominally antiviral products, how effective they were remains even now to be analysed and documented. Yet, the continued prevalence of SARS-CoV-2 despite the widespread adoption of face masks, hand sanitisers, and elbow bumping could provide anecdotal evidence that these measures were not as effective as public health authorities thought at the time [19].

## 7 Separating prevention practices that are distinct to nations, societies and cultures

In the emergency response to COVID-19, ostensibly the entire world adopted similar practices, even though geographic and demographic circumstances, cultural practices and societal implementation varied [34]. In high-income countries, it was common for a residence to be occupied by a single person, while overpopulated domiciles were rare. However, this is not the case with sub-Saharan African homes in which it is the cultural norm for at least three to four people to share the same space across urban and rural communities. Therefore, asking people to stay at home may prevent the spread of infection in the Global North but allow continued disease transmission in the Global South [9].

In contrast, while windows are hardly ever opened in industrialised nations in temperate climates, the heat and humidity of Africa make the opening of windows a preferred lifestyle choice. This means that typically without the installation of air conditioning units, homes in sub-Saharan Africa have access to natural ventilation, the effect of which is to dilute the interior concentration of respiratory infective agents [9]. On the other hand, windows in high-income countries were hardly ever opened, allowing stale air to be recirculated in residential and office buildings, thereby increasing potential transmission [9]. This makes the stay-at-home policy debatable in both cultural contexts and may account for the transmission of SARS-CoV-2 among those who self-isolated as instructed.

Future pandemic policies should acknowledge the continent's great experience with containment of other viral illnesses and allow the development of local protocols that suit individual nations, rather than allowing a “one-size-fits all” approach mandated by Europe or the United States. It could be argued that with a younger population than other continents, the need for lockdown was less important than in the Global North and that the development of herd immunity might have been more sensible [9]. More detailed studies should be conducted to determine the efficacy of the stay-at-home policy across the world.

## 8 Discussion

With the benefit of hindsight, the global health response to COVID-19 could have learned much from the 1918–1919 “Spanish” influenza pandemic in terms of lockdown, mask-wearing and other containment strategies. Regionally, other disease outbreaks in Africa could have been scrutinised more closely, such as the multiple Ebola recrudescences and even animal viral infections like the great 1888 rinderpest epidemic, where quarantine policies were first promulgated. Unfortunately, however, in the panic that beset the world in early 2020, these historical precedents were mostly overlooked, so vanishingly few of the proven practices from the past were applied [[35], [36], [37], [38]].

Nevertheless, COVID-19 has provided insights that are critical to enhance the management of future pandemics in sub-Saharan Africa. The impact of the pandemic on children in the region underscores the need to prioritise preventive health services, including vaccination and malaria control, in order to safeguard vulnerable populations [39]. In order to ensure an effective response, it is crucial to allow for proper training of healthcare professionals with future locally-designed containment strategies while addressing burnout and supporting frontline workers, even in the most remote areas. Additionally, the high seroprevalence among emergency responders in urban settings highlights the urgency of developing locally parameterised mathematical models to predict epidemic trajectories for evidence-based policy decisions and public health response planning [40].

Genetic variations in sub-Saharan Africa may confer resistance to COVID-19, suggesting the importance of understanding regional genetic factors in pandemic management [41]. Furthermore, the pandemic has underscored the imperative for sub-Saharan Africa to build capacity for manufacturing vaccines, therapeutics, and diagnostics to address public health crises effectively [42]. Lessons from recent Ebola epidemics have offered valuable awareness of how best to strengthen the COVID-19 response in the region, emphasising the need for effective translation of these lessons into pandemic management strategies [43].

It is important to stress that community-based and community-led strategies are crucial for achieving pandemic control in sub-Saharan African communities, necessitating the availability of necessary socioeconomic resources and contextual adaptation of interventions [44]. Future pandemic strategies can only succeed if there is a grassroots approach. Moreover, the pandemic has revealed the importance of so-called ‘systems thinking’ in COVID-19 recovery to deliver sustainable development for African women and girls [45]. However, challenges such as poorly resourced mental health systems, gender inequalities, and the impact on adolescent health and well-being need to be addressed to improve pandemic preparedness on the continent.

These lessons collectively provide a comprehensive framework for enhancing future pandemic preparedness and response efforts in sub-Saharan Africa, encompassing various aspects of healthcare, public health, community engagement, and sustainable development [46].

## 9 Conclusions

Reflecting on how the COVID-19 pandemic was handled by public health authorities across the globe but especially in sub-Saharan Africa, there is much that can be learnt from history, both recent and from long-distant pandemics, in preparing for emerging and re-emerging infectious disease outbreaks in coming years [47]. We argue for a reasoned and informed approach on the part of those in power. In the often-authoritarian modes of governance prevalent on the continent, many governments have been unused to formulating policy in anything other than through diktat. Having panels of independent expert medical advisors informing policymakers at the highest level would obviate many of the uninformed measures that were rapidly adopted in the early phases of the COVID-19 pandemic. Here, we have identified four issues that should be addressed.

1) Separating science from politics is a complex and multifaceted issue. On the one hand, the integrity of scientific research and evidence-based decision-making is crucial for addressing public health challenges effectively. On the other hand, however, the intersection of science and politics is inevitable, particularly in matters of public policy, resource allocation, and regulatory frameworks. While it is important to maintain the autonomy and objectivity of scientific inquiry, it is equally important for policymakers to consider scientific evidence when formulating policies [47]. We acknowledge that this is far from straightforward to achieve, but governments across Africa should be encouraged to harness independent expert advice for the greater good of their populations. We believe that the African Union (AU) should play a role in fostering the right climate of governance through developing further governmental charters on health policy.

2) Separating medical practice from business is a critical consideration in ensuring the ethical delivery of healthcare. While healthcare is undoubtedly a business in terms of resource allocation and financial sustainability, the primary focus should always be on patient care and well-being. The commercial aspects of healthcare should not compromise the quality of medical services or patient outcomes. It is essential to maintain the integrity of medical practice by prioritising evidence-based care, patient safety, and ethical decision-making, rather than solely focusing on financial gain. This separation is crucial for upholding the trust and confidence of patients and the community in the healthcare system. Additionally, it is important for healthcare professionals to adhere to ethical guidelines and standards, ensuring that patient care remains the central focus of medical practice. Again, these ideals are challenging to achieve, but the AU should take a lead in setting standards across the continent.

3) Separating proven public health practices from untested behaviours is essential for safeguarding the well-being of communities. Evidence-based public health practices are rooted in rigorous research, empirical data, and scientific consensus, providing a foundation of reliability and effectiveness. In contrast, untested practices may lack empirical support and could potentially pose risks to public health. In the advent of the multimedia era, public education programmes should utilise both conventional communication channels and social media strategies to reach all demographic groups in each community. It is crucial to prioritise evidence-based interventions and policies to ensure the safety and effectiveness of public health initiatives. This approach requires a comprehensive evaluation of available evidence and a commitment to implementing practices that have demonstrated positive outcomes in public health. Apart from the AU and regional political and economic organisations, such as the East African Community, standards should be set and upheld by peak professional bodies, including the West African College of Physicians (WACP) and the East, Central and Southern African College of Physicians (ECSACOP).

4) Separating prevention practices that are unique to nations, societies, and cultures. This is a complex and multifaceted issue that involves understanding the local social, cultural, and contextual factors that influence health behaviours and practices. Public health strategies should be sensitive to cultural norms, values, and beliefs, and should be designed to be culturally appropriate to increase their relevance and effectiveness. However, it is important to critically evaluate these practices to ensure that they are evidence-based and aligned with public health goals. Understanding the social and cultural context is essential for tailoring prevention strategies to specific populations, but it is equally important to ensure that these strategies are grounded in scientific evidence and contribute to positive health outcomes. While it may be difficult to hold individual countries to account, pan-African and African regional political, economic and medical organisations, ranging from the AU to ECSACOP, may drive monitoring of key issues.

It is to be hoped that by implementing these ideas, low-income countries in sub-Saharan Africa will be far better resourced, equipped, and prepared to combat the next infectious disease epidemic or pandemic than our lived experiences tell us they were for COVID-19. If ever there was a case of wishing we had known then what we know now, this is it. The imperative is to learn from our previous mistakes, not to let history repeat itself.

## Authors’ contributions

Article conception, OOO; literature search and data collection, OOO and AWT-R; interpretation of information, all authors; writing — original draft preparation, OOO and AWT-R; writing — manuscript preparation, all authors; writing — editing critically for important intellectual content, SDT-R. All authors read and approved the last version of the manuscript.

## Ethical approval

Not required.

## Funding

SDT-R is grateful to the United Kingdom National Institute for Healthcare Research at 10.13039/501100000761Imperial College London for infrastructure support. He is funded in part by a 10.13039/100010269Wellcome Trust Institutional Strategic Support Fund grant awarded to 10.13039/501100000761Imperial College London (grant number 105603/Z/14/Z).

## Declaration of competing interest

The authors declare that they have no known competing financial interests or personal relationships that could have appeared to influence the work reported in this paper.
